# A Novel Persian Herbal Syrups: Preventive and Curative Effects of Syrup Formulation of Achillea. millefolium L. against Ethylene Glycol Induced Urolithiasis in Rats

**DOI:** 10.31661/gmj.v13i.3317

**Published:** 2024-09-17

**Authors:** Mozhgan Dahmardnezhad, SeyedMahmoudReza Hashemi Rafsanjani, Sara Sabbaghi, Nina Baghinia, Nastaran Fooladivanda, Elham Peyravi, Mahmoud-Reza Baghinia, Yekta Parsa, Hossein Ghasemzadeh Kolagar, Fardad Saremi, Zahra Abbasy, Seyyed Mojtaba Ghorani, Sahar Rezaei Nezhad Rafsanjani, Hamid Zaferani Arani

**Affiliations:** ^1^ School of Medicine, Arak University of Medical Sciences, Arak, Iran; ^2^ Young Researchers and Elite Club, Tehran Medical Sciences, Islamic Azad University, Tehran, Iran; ^3^ School of Pharmacy and Pharmaceutical Sciences, Tehran Medical Sciences, Islamic Azad University, Tehran, Iran; ^4^ Department of Pathology, Shiraz University of Medical Sciences, Shiraz, Iran; ^5^ Department of Surgery, Shiraz University of Medical Sciences, Shiraz, Iran; ^6^ Department of Urology, Faculty of Medical Sciences, Arak University of Medical Sciences, Arak, Iran; ^7^ Department of Obstetrics and Gynecology, Shahid Beheshti University of Medical Sciences, Tehran, Iran; ^8^ Health Technology Incubator Center, Shahroud University of Medical Sciences, Shahroud, Iran; ^9^ Department of Marine science, Science & Research Branch, Islamic Azad University, Tehran, Iran; ^10^ Department of Paediatrics, Tehran University of Medical Sciences, Tehran, Iran

**Keywords:** Herbal Extracts, Achillea Millefolium, Ethylene Glycol, Kidney Stones

## Abstract

Background: The urinary system is afflicted by urolithiasis, which stands as the third most common disabling disorder. The application of herbal plants is a widespread practice there is an increasing interest in research in this domain to establish scientific reasons for their beneficial properties. Hence, this study aimed to investigate the protective effects of the novel syrup formulations of Achillea millefolium against urolithiasis. Materials and Methods: The two suspensions of A. millefolium, i.e., ethanolic and aqueous extracts were prepared. The 36 male Wistar rats were divided to six groups, i.e., group A (control), and ethylene glycol (EG) 1%-induced nephrolithiasis (groups B to F). The curative (C and D) and preventive (E and F) groups received 300 mg/kg body weight extracts orally from day 15 and first, respectively. After 28 days, the serum and urine samples, as well as, kidneys were taken for analysis and histopathologically for counting the calcium oxalate (CaOx) deposits, respectively. Results: Serum parameters such as creatinine, blood urea nitrogen, and uric acid of group B rats were increased significantly in comparison to normal rats (P0.001) and extracts-treated groups (C to F). Also, our results indicated marked (P0.05) reductions in urinary oxalate, phosphate, and calcium in A. millefolium-treated rats; however, urine citrate in rats of group B was significantly reduced compared with other groups. Also, compared with group B, histopathological examinations revealed CaOx deposit reduction in groups C to F (P0.05). Conclusion: Results of our study show that the treatment of rats with A. millefolium extracts had curative, as well as preventive properties on EG-induced kidney stones.

## Introduction

Urolithiasis is the third common chronic disorder of the urinary system and its incidence is estimated from 2% to 20 % in the Middle East countries [1]. Near 80% of kidney stones are composite of calcium, oxalate, and calcium phosphate [2]. Currently, extracorporeal shock wave lithotripsy, local calculus disruption using laser, and medication therapy are widely used against urolithiasis [3]. However, these procedures are highly costly and may lead to serious complications such as hemorrhage, decrease in renal function, and hypertension [4][5]. Therefore, the use of herbal plants can be replaced with conventional treatments to reduce these side effects [6][7]. Increasing interest in the use of medicinal plants around the world, as well as research and scientific fields for their beneficial effects, have also been performed [7].

Achillea millefolium L., (named Bumadaran in the Persian language) is a genus of the Asteraceae family that is found in Europe, Asia, and North America [8], and in Iran, it grows in the northern areas in some provinces, including Azerbaijan, Lorestan, and Isfahan [9]. A. millefolium has long been used in traditional Persian medicine. A. millefolium L. was first mentioned in the Makhzan-ol-Advieh book written by Aghili-Shirazi in the 18th century [10]. Previous evidence reported anti-hemorrhoids, anti-inflammatory, and wound healing effects for A. millefolium [9][10]. In addition, other activities of A. millefolium were mentioned in traditional medicine books, including anti-bacterial, astringent, anti-oxidative, hepatoprotective, anti-spasmodic, anti-dysenteric, antipyretic, diuretic, anti-urolithiasis, urinary anti-septic by native peoples [11][12]. Also, its anti-nociceptive and calcium-antagonist activities are beneficial in the prevention of urolithiasis [13][14]. Previously, in the pilot study, we reported the beneficial effect of hydroalcoholic extract of A. millefolium on kidney stone formation [15]. However, the solubility of the active substance varies in the aqueous and ethanolic phases; hence, the aim of this study was to evaluate two novel A. millefolium syrups with different formulations for preventive and curative effects on urolithiasis animal models.

## Materials and Methods

Animals

This manuscript has been done in two steps, in the first step, 16 rats were used to perform acute toxicity, and in the second step, 36 rats were used to perform animal trials. Thirty-six male Wistar rats (200-250 g) were purchased from the Karaj branch of Pasteur Institute (Iran) and kept in standard cages under 25±2 ◦C with 12/12 hours of light/dark cycles for a week. and fed with a standard pelleted diet and water.

Plant Collection and Identification

The aerial parts of A. millefolium were obtained from northwest Iran and identified by the Pharmacognosy Department of Tehran University recognized it with herbarium number: 83001. The plants were then dried and powdered in a standard manner.

Preparation of Plant Extracts

The dried A. millefolium was pulverized and extracted with 95% ethanol for a total of seven hours, followed by a 1.5 hour extraction with distilled water. The resulting ethanol and water extracts were filtered, concentrated, and dried in an oven at 50◦C. The ethanolic extract yielded 9.2% and the aqueous extract yielded 13.41%. The extracts were refrigerated at four degrees Celsius and diluted with distilled water before being tested [16].

Preparation of Suspension-I Formulation

Six g of the dried, powdered ethanolic extract of A. millefolium was suspended in water using 6g of Arabic gum (Sigma-Aldrich, Germany) as a suspending agent. Then 0.2 g and 0.06 of methyl paraben and propyl paraben (Sigma-Aldrich, Germany) were added as additive agents, respectively.

Preparation of Suspension-II Formulation

Six g of the dried, powdered aqueous extract of A. millefolium was triturated by using a mortar and pestle and it was added 1g sodium carboxymethyl cellulose (Sigma-Aldrich, Germany) as a suspending agent then made volume by sugar syrup. Also, 0.05 g, 0.06 g, and 0.2 g of Amaranth (Sigma-Aldrich, Germany), methyl paraben, and propyl paraben were added as coloring and additive agents, respectively.

Acute Toxicity

The acute toxicity study followed guidelines from the Organization for Economic Cooperation and Development (OECD) and the Committee for the Purpose of Control and Supervision of Experiments on Animals (CPCSEA) [12], using healthy Wistar rats of both sexes weighing 200-250 g. The rats were divided into two groups of 8, with each group receiving different doses. The rats were deprived of food overnight but had access to water. They were then given two different doses of oral suspensions, 1500 and 3000 mg/kg of body weight, and observed for 24 hours with no negative effects or fatalities. According to OECD guidelines 420, the median lethal dose (LD50) was determined to be greater than 3000 mg/kg, so one-tenth of that dose was used for the anti-urolithiasis treatment [12].

Experimental Groups

Rats were grouped randomly (n=6 per group) as follows:

Group A that considered as control rats and received only regular food and drinking water ad libitum. Groups B to F were fed ethylene glycol (EG) 1% (Merck, Germany) by the use of drinking water to induce nephrolithiasis [15] in rats from the first day till the 28th day. Group B was considered as sham group and received no any treatments. Groups C and D (curative groups) received 300 mg/kg b.wt Suspension-I and -II from days 15 to 28, respectively. Groups E and F (preventive groups) received Suspension-I (300 mg/kg b.wt) and suspension-II (300 mg/kg b.wt) from the first to 28th day, respectively [15]. Rats were fed with extracts once daily via oral route.

Urine Analysis

Urine-24-hour samples were collected using standard metabolic polypropylene cages at the end of the study. The urine volume of each rat was calculated for each group and a drop of hydrochloric acid was added to the collected urine before being stored at 4◦C. Urinary parameters (such as oxalate, calcium, phosphorus, and citrate) were measured by commercial kits (Darman Kaw, Tehran, Iran) with an auto-analyzer (SB 501 Plus, Sinduri Biotec, India).

Blood Sample Collection

At the conclusion of the experiment, blood was obtained through retro-orbital puncture while the animal was under ether anesthesia. The serum was then separated by centrifugation at 2500 rpm for 10 minutes [15].

Serum Analysis

The levels of serum creatinine, blood urea nitrogen, and uric acid were measured using kits and a spectrophotometer from MAN, Tehran, Iran, following the manufacturer’s instructions. The concentrations were calculated using the formula (mg/dl)=[(Ablank- Asample)/Ablank] where Ablank represents the absorbance of the control reaction (containing all reagents except the test compound) and Asample represents the absorbance of the test compound. The results were reported in mg/dl.

Histopathology Examination

On the 28th day’s conclusion, anesthesia was administered to all the rats, followed by decapitation using a guillotine. Subsequently, both kidneys were extracted and preserved in formalin (10%) for histological investigations. From each kidney, three 5µm sections were meticulously prepared, and the resulting slides were subjected to staining with hematoxylin-eosin (H&E). These stained slides were then examined under a light microscope. The number of calcium oxalate (CaOx) deposits in the renal tubules was quantified by counting them in 10 microscopic fields.

Ethics Statement

This study and all protocols were performed based on the Principles of Laboratory Animal Care (NIH publication, 9th edition). Also, the Ethics Committee of Islamic Azad University, Tehran Medical Sciences Branch, Tehran, Iran approved the current study via number: 12/4/3109.

Statistical Analysis

The data was expressed as the mean±SD and analyzed using SPSS version 14 (SPSS Inc, Chicago, IL, USA) with one-way ANOVA and Tukey post hoc test for multiple comparisons. A significant difference was determined at P<0.05.

## Results

**Table:** Table1. Effect Of Plant Extracts On Urinary Biochemical Parameters On The 28th Day

**Groups**	**Oxalate** **(mg/dl)**	**Calcium** **(mg/dl)**	**Citrate** **(mg/dl)**	**Phosphate** **(mg/dl)**
**A**	0.79±0.04	1.73±0.03	19.23±0.03	3.19±0.05
**B**	4.11±0.06**	3.5±0.15**	7.27±0.02**	7.2±0.01**
**C**	2.61±0.01*	2.42±0.15*	10.16±0.07*	5.25±0.03*
**D**	2.04±0.09*	2.7±0.03*	11.21±0.02*	5.59±0.1*
**E**	2.5±0.01*	2.55±0.03*	10.25±0.01*	5.26±0.03*
**F**	1.79±0.02*	2.57±0.02*	11.34±0.07*	5.51±0.01*

^*^ P<0.05, Achillea groups vs. groups A and B ^**^ P<0.05, group A vs. group B

A. millefolium Administration Could Improved Urine Parameters Against Urolithiasis

There was a marked increase in oxalate, calcium, and phosphate excretions in the sham group (group B) in comparison to control rats (P<0.05, Table-1). However, administration of A. millefolium significantly prevented these changes in urinary oxalate, calcium, and phosphate excretion in groups C to F compared to group B (P<0.05). As shown in Table-1, there was a significant difference between A. millefolium-treated groups in terms of oxalate (P<0.001).

The mean urinary citrate level in group A (control rats) was 19.23±0.03 mg/dl, while citrate excretion was significantly decreased by urolithiasis to 7.27±0.02 mg/dl (Table-1, P=0.011). However, administration of A. millefolium significantly prevented these changes (P<0.001). Also, citrate changes between A. millefolium-treated groups were significant (Table-1, P<0.01). Based on the results of urine analysis, among A. millefolium-treated groups, rats in group F had significantly lower oxalate and higher citrate in comparison to group B (P=0.018 and P=0.003, respectively).

Serum Cr, BUN, and Uric Acid Concentrations

Regarding Table-2, the mean Cr concentration in groups A and B was 0.49±0.03 and 2.57±0.06, respectively. Indeed, serum Cr was increased significantly following urolithiasis; however, A. millefolium administration significantly reduced the serum Cr in group C to F in comparison to the sham group (Table-2, P<0.001).

Also, the mean BUN and uric acid concentrations of the rats in the sham group were significantly higher than those of all other groups (Table-2, P<0.05). However, those concentrations in the curative and preventive groups were significantly decreased in contrast to the sham group (Table-2, P<0.01).

CaOx Deposits

**Figure-1:**
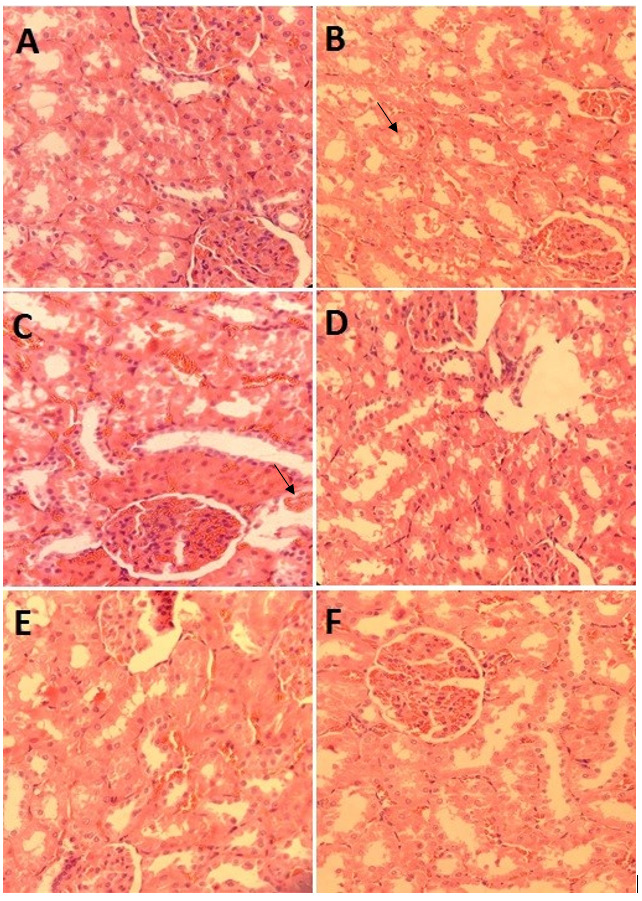
Figure 1. The microscopic histology of the kidney sections. A: Control group without any CaOx deposition; B: EG group showed the most CaOx deposition (arrow); C and D: In the curative groups, CaOx depositions (arrow) significantly reduced compared to EG group; E and F: Preventive groups indicate the low CaOx depositions compared to other groups (hematoxylin-eosin stain, × 400).

Based on histopathological examination, no CaOx deposits were seen in the nephron segment of rats in the control group. Indeed, many CaOx deposits were observed in the rats of groups B to F (Figure-1). Regarding Table-3 , the mean CaOx deposits in groups C and D were 18.17±0.69 and 15.5±0.76, respectively, which was significantly lower than that in group B (P=0.03 and P=0.002, respectively). In addition, in the preventive groups (E and F) the CaOx deposits were significantly lower than those in group B (Table-3).

## Discussion

**Table:** Table2. Effect Of Plant Extracts On Serum Biochemical Parameters On The 28th Day

**Groups**	**Uric acid** **(mg/dl)**	**Creatinine** **(mg/dl)**	**BUN** **(mg/dl)**
**A**	1.62±0.03	0.49±0.03	23.17±1.34
**B**	4.24±0.03**	2.57±0.06**	33.67±1.11**
**C**	2.89±0.01*	1.77±0.01*	16.5±0.96*
**D**	2.64±0.01*	1.43±0.01*	15.5±1.26*
**E**	2.75±0.04*	1.66±0.03*	21.5±0.96*
**F**	2.38±0.02*	1.12±0.03*	18.5±0.96*

^*^ P<0.05, Achillea groups vs. groups A and B ^**^ P<0.05, group A vs. group B

**Table:** Table3. Effect of Plant Extracts On Calcium Oxalate Deposition In The Kidneys Of Rats On The 28th Day

**Parameter**			**Groups**			
	**A**	**B**	**C**	**D**	**E**	**F**
**CaOx deposition**	-	33.67±1.11**	18.17±0.69*	15.5±0.76*	8.83±0.89*	5.83±0.69*

^*^ P<0.05, Achillea groups vs. groups A and B ^**^ P<0.05, group A vs. group B

In the current study, the effects of two formulations of A. millefolium were evaluated in prevention as well as treatments of urolithiasis in rat models. Urinary parameters, including oxalate, Cr, and phosphate were significantly increased after EG-induced urolithiasis, while citrate level was reduced. A. millefolium administrations could significantly show both preventive and treatment effects. Also, serum parameters changes in rats that received A. millefolium syrups markedly improved against control and sham groups. In addition to urine and serum biochemical parameters, the histopathological study revealed the beneficial effects of A. millefolium in CaOx deposit prevention.

In traditional medicine, A. millefolium was administered usually through the oral route [10]. Hence, in our study, the same route was taken for evaluating the anti-urolithiasis effect of the A. millefolium in the rat model. Previous evidence indicates that the rate of kidney stone formation in male rats, as in humans, was higher than the females. Hence, male rats were used to induce urolithiasis in the present study [15][17].

Previous studies have provided evidence suggesting that the administration of EG to young rats for a duration of two weeks led to the formation of renal calculi primarily composed of CaOx [18][19]. The development of nephrolithiasis in EG-fed animals can be attributed to hyperoxaluria, resulting in the increased retention and excretion of oxalate in the kidneys. [17][20][21]. Therefore, this model was used to evaluate the protective effect of A. millefolium syrups against urolithiasis.

In the current study, urinary oxalate and calcium excretions were increased in EG-induced urolithiasis rats. Also, a reduction in oxalate and calcium was observed in A. millefolium treated groups. The plant extract may have contributed to the decrease in oxalate excretion by inhibiting oxalate formation. Rats with calculi, specifically in group B without A. millefolium syrups displayed a notable reduction in urinary citrate levels.

Some evidence has shown that the reabsorption of citrate in tubules plays a regulating role by forming a complex with calcium thereby reducing the concentration of CaOx [22][23][24]. In our study, it was found that A. millefolium treatment brought the urinary citrate excretion level near to its level among control rats to decline the risk of stone formation.

In rats with EG-induced urolithiasis, there was an increase in the amount of phosphorus excreted in their urine. This, along with oxalate, has been linked to the formation of stones through the formation of calcium phosphate crystals which induces CaOx deposition [17][22][25]. However, treatment with A. millefolium reduced phosphorus excretion and the risk of stone formation, while the administration of an aqueous extract-based suspension decreased oxalate and increased citrate compared to other treatment groups.

The accumulation of waste products, particularly nitrogenous substances such as Cr, BUN, and uric acid, in the blood is a consequence of the decreased glomerular filtration rate (GFR) observed in urolithiasis [26][27][28]. The current study reveals that nephrotoxicities induced by EG are characterized by a notable rise in serum Cr, BUN, and uric acid levels. The administration of both suspensions significantly mitigated nephrotoxicity, as evidenced by an improvement in GFR and a subsequent reduction in the presence of nitrogenous waste products. While there was no significant difference between the two suspensions.

Microscopic evaluation of renal sections obtained from rats with calculi revealed the existence of polymorphic and irregular crystal deposits inside the tubules. These deposits resulted in the enlargement of the proximal tubules and the development of interstitial inflammation, which could potentially attributed to the presence of oxalate. Nonetheless, the administration of A. millefolium suspensions exhibited a significant reduction in both the quantity and dimensions of CaOx deposits in various regions of the renal tubules. So, results showed the protective effect of A. millefolium syrups with ethanolic and aqueous bases in the EG-induced urolithiasis model.

The previous study [15] reported high antioxidant and anti-inflammatory effects of A. millefolium. It can be speculated that the anti-urolithiasis formation activity of the kidney may be through of oxidant activity and free radicals. In other words, the administration of ethanolic and aqueous extracts of A. millefolium reduces and prevents the growth of urinary stones. However, better protective effects were observed following the administration of A. millefolium aqueous extract from the first day of the study.

This study has some limitations. The mechanism underlying the anti-urolithiasis effect of A. millefolium was not exactly evaluated, but it could related to diuresis and the lowering of urinary concentrations of stone-forming constituents. Also, in this study we used the whole of A. millefolium extracts; however, the main bioactive components were not evaluated. Hence, further studies are still recommended on the various aspects and side effects of this herbal agent.

## Conclusion

Our findings indicate that the use of both A. millefolium formulations showed preventive and curative effects on EG-induced urolithiasis, providing further support for the traditional use of A. millefolium in treating urolithiasis.

## Conflict of Interest

The authors report no conflicts of interest.
